# Direct and indirect savings from parallel imports in Sweden

**DOI:** 10.1186/s13561-022-00391-x

**Published:** 2022-08-31

**Authors:** David Granlund

**Affiliations:** Department of Economics, Umeå University, Umeå, Sweden

**Keywords:** Brand-name drugs, Parallel trade, Pharmaceutical industry, Pharmacies, Price competition

## Abstract

**Background:**

The aim was: i) to quantify the direct and indirect savings from parallel imports in Sweden during a period when sellers were forbidden from giving discounts to pharmacies, and ii) to study if the effects of competition from parallel imports on list prices became smaller in absolute size when sellers were allowed to give discounts to pharmacies.

**Methods:**

We analyzed the monthly prices for 3068 products during 61 months when discounts were forbidden and for 2504 products during 84 months when discounts were allowed. The price effects were estimated using dynamic models that rendered lagged numbers of competitors into valid and strong instruments for the current values.

**Results:**

When discounts were forbidden, parallel imports had a market share of 16% and were on average 9% cheaper than locally sourced drugs, which yielded a direct saving of 231 million Swedish kronor (SEK) (24 million EUR) per year. Also, parallel imports reduced the prices of products with the same substance by, on average, 6% in the long-term, which yielded indirect savings of 421 million SEK (44 million EUR) per year. In total, parallel imports reduced the cost for on-patent pharmaceuticals by 4%. When discounts were allowed, the average gap in list price between parallel imports and locally sourced products was reduced to 0.8%, and the list prices of locally sourced products were no longer significantly affected by competition from parallel imports.

**Conclusion:**

When discounts were allowed, the savings of parallel imports through lower list prices were replaced by savings of pharmacies through secret discounts.

## Background

In an attempt to practice third-degree price discrimination, producers may charge wholesalers in low-income countries less than they charge wholesalers in high-income countries. Parallel traders take advantage of these price differences by buying products intended for low-price countries and, without authorization from the patent holder, selling them to wholesalers in high-price countries. Parallel trade is allowed within the European Economic Area to help fulfill the objective of creating a single market.

This paper evaluates the savings from parallel imports in Sweden during a period when sellers of pharmaceuticals were forbidden from giving discounts to pharmacies. This situation implied that the official list prices were actual transaction prices, which enabled us to quantify the total savings. We also studied whether the effects of competition from parallel imports on list prices became smaller in absolute size when sellers were allowed to give discounts to pharmacies. There are several reasons why firms might prefer to give discounts, rather that lower official list prices, to reduce the market share of parallel imports. Lower list prices, for example, can reduce the revenues from other countries where the Swedish list prices are used as external reference prices,^1^ and, in Sweden, list prices might not be allowed to be increased within the benefit scheme when the competition diminishes.

With access to data on market shares for parallel imports and relative prices, direct savings can be easily calculated. This has been done previously, for example, by West and Mahon [2], who reported direct savings for Sweden of 424 million Swedish kronor (SEK) in 2002 (measured in retail prices).

West and Mahon [2] also showed price plots and comparisons of price changes over 5–6 years, which indicated that parallel imports exerted downward pressure on prices. Estimating this effect is difficult, however, because parallel imports are more likely to be sold the higher the prices of the locally sourced products are, rendering the variable endogenous. To address this endogeneity problem, Ganslandt and Maskus [3] and Granlund and Köksal-Ayhan [4, 5] used exchange rates and the age of drugs as instruments for competition from parallel imports and reported point estimates suggesting that competition from parallel imports reduced the prices of locally sourced drugs in Sweden by 12 to 21%. However, these instruments may affect the prices of locally sourced drugs in other ways than through the existence of parallel imports, which can create bias. For example, with a stronger Swedish currency, a producer can reduce the nominal price in Sweden without having to reduce the price in countries where the maximum allowed prices depend on Swedish prices measured in Euros.

Vandoros and Kanavos [6] instead used instruments based on the number of policies promoting parallel imports and the distance between the source countries and the four destination countries they analyzed (Germany, Sweden, the Netherlands, and the United Kingdom). They found no statistically significant price effect, but because of large standard errors, they could neither reject the premise that the price effect was large.^2^ Vandoros and Kanavos also analyzed the effect of the market share of parallel imports—following Kanavos and Costa-Font [7] and Kanavos and Vandoros [8]—but, like the previous studies, they found no statistically significant price effects. Christian Gollier, the discussant to Kanavos and Costa-Font, mentioned as one potential explanation for the lack of a significant estimate the possibility that “the local manufacturer actually matches the price of the importers by using hidden discounts to distributors rather than reducing the list price” [6 , p. 793].

To overcome the problem with weak and potentially endogenous instruments, Granlund [9] used a dynamic model that allowed lags of competition variables to be used as instruments for their current values. This approach yielded sufficiently many strong instruments for also studying the causal effects on the intensive margins, i.e., how the number of parallel traders and the number of therapeutic competitors affect prices. Granlund [9] used part of the data used in the present study: that for tablets and capsules sold in October 2002–October 2007. For this study, we estimated similar price functions as Granlund [9], but also did so for the period of January 2011–December 2017, for all forms of administrations, and calculated the direct and indirect savings yielded by parallel imports.

## Rules regarding parallel imports

All Swedish residents are covered by a mandatory and uniform pharmaceutical benefit scheme. Since October 2002, a substitution legislation requires that pharmacy personnel inform consumers if cheaper substitute products are available, unless the prescriber has vetoed substitution or if the pharmacist has reasons to believe that the patient would be adversely affected, e.g., when the low-cost alternative has a package that the patient would find difficult to open. The Swedish Medical Products Agency defines a product as a substitute if it has the same active substance, strength, and form of administration (e.g., pills or oral fluid) and nearly identical package size.^3^ If consumers oppose the substitution, or choose to switch to a substitute other than the cheapest one available, they will be charged the entire incremental cost. For parallel imports, available substitutes are defined as those in stock at the pharmacy in question [10].^4^

Pharmaceutical producers and parallel traders are free to set their own prices, but to be included in the pharmaceutical benefits scheme, they must submit their prices for month *t* to the Pharmaceutical Benefits Agency (PBA) in month *t – 2*. The PBA approves prices not exceeding the price cap, which is equal to the highest existing price of exchangeable products, which implies that parallel imports are allowed to be as highly priced as locally sourced products [11, 12]. The price cap may prevent the seller of a product that is already the most expensive among its substitutes from increasing the price, if they want their product to remain within the benefit scheme. This means that a price cut retrospectively found to be too large cannot always be reversed. Before July 2009, producers and parallel importers were not allowed to offer their products below the prices approved by the PBA prices. That is, they were not allowed to give discounts to pharmacies.

## Methods

### Data

This study was based on two panel datasets obtained by merging datasets of pharmaceutical sales compiled by IMS Sweden (now part of IQVIA) with datasets containing detailed information of each pharmaceutical product, which were provided by the Västerbotten county council.^5^ An observation in the datasets represents a product with a certain active ingredient, strength, administrative form, and package size, supplied by a certain firm and sold in a certain month. The datasets cover all prescription drugs sold in Sweden during the periods of October 2002–October 2007 and January 2011–December 2017. Data from November 2007–June 2009 were not used because prices during this period could have been affected by anticipation of the possibilities of giving discounts. Because price increases are not always allowed within the benefit scheme, it would have been rational for firms to stop reducing list prices in response to competition upon discovering the possibility of giving discounts to pharmacies in the future. In this manner, they would have had higher list prices and therefore greater possibilities to give discounts upon legalization of the practice, compared to if they continued reacting to competition with lower list prices until the day discount was legalized.^6^ We also excluded data from July 2009–December 2010, as the business models related to discounts might still have been under rapid development under this period. Lacking information on patent expiration, we defined pharmaceuticals as off-patent starting from the first time any generics with the same active ingredient (i.e., the same 7-digit ATC code) were sold in Sweden, and pharmaceuticals are included in the analyses until the month they are designated to be off-patent. After excluding off-patent pharmaceuticals, the first and second datasets respectively contained 132,008 and 101,489 observations of locally sourced product and 31,999 and 70,540 observations of parallel-imported products. That is, for an average year, the first and second datasets respectively contained 25,969 and 14,498 observations of locally sourced products and 6295 and 10,077 observations of parallel-imported products.

### Estimation of price effects and descriptive statistics

For several reasons, prices are not expected to adjust instantaneously to new long-term equilibriums when market conditions change. One reason is possible price coordination between therapeutic alternatives, which can cause companies to limit price changes to reduce the risk of triggering price wars [14]. Another reason is the dynamic price cap on drugs in Sweden, which means that a drug whose price is raised to a figure higher than that of the most expensive substitute can be excluded from the pharmaceutical benefit scheme. A company that is uncertain about what the new optimal price is after it has received competition may, because of this price cap, find it wise to lower the price gradually, rather than to lower it more directly and then risk not being able to adjust the price if it is found that the price cut was unnecessarily large. For these reasons, we estimated price effects with dynamic models.

The preferred specification, which was estimated with two-stage least squares using the STATA package xtivreg2, is written as:

$${\displaystyle \begin{array}{c}{lnP}_{it}=\theta {lnP}_{i,t-1}+{\beta}_1D\_{PiSubstance}_{st}+{\beta}_2D\_{PiE}_{it}+{\beta}_3 lnN\_{PiSubstance}_{st}+{\beta}_4 lnN\_{PiE}_{it}\\ {}+{\beta}_5D\_{Th}_{st}+{\beta}_6D\_{Th Gen}_{st}+{\beta}_7 lnN\_{Th}_{it}+{\beta}_8 lnN\_{Th Gen}_{st}+{\eta}_t+{\mu}_i+{\varepsilon}_{it},\end{array}}$$

in which indices *i*, *s*, and *t* represent product, substance, and time in months, respectively. Variable definitions are presented in Table 1 and in the text below. The dependent variable *lnP*_*it*_ is the natural logarithm of the listed purchase price for all pharmacies for the on-patent locally sourced product *i* in month *t*. The first lag of this variable, *lnP*_*i*, *t* − 1_, was included as an explanatory variable to make the model dynamic.

**Table 1:** Variable definitions

*lnP*_*it*_	Natural logarithm of the listed purchase price for product *i* in month *t*.
*D* _ *PiSubstance*_*st*_	Equals one if one or more parallel imported product with the same active substance as product *i* were sold in Sweden in month *t*.
*D* _ *Pi*_*it*_	Equals one if one or more parallel imported product exchangeable with product *i* was sold in month *t*.
*N_PiSubstance*_*st*_	The number of parallel traders that sold products with the same substance as product *i* in month *t*.
*lnN_PiSubstance*_*st*_	Natural logarithm of *N_PiSubstance*_*st*_ when *N_PiSubstance*_*st*_ > 0, but equal to zero when *N_PiSubstance*_*st*_ = 0.
*N*_ *Pi*_*it*_	The number of parallel traders that in month *t* sold products exchangeable with product *i*.
*lnN* _ *Pi*_*it*_	Natural logarithm of *N_Pi*_*it*_ when *N_Pi*_*it*_ > 0, but equal to zero when *N_Pi*_*it*_ = 0.
*D* _ *Th*_*st*_	Takes the value of one if one or more other firm in month *t* sold a locally sourced product with the same five-digit ATC code as product *i*.
*D* _ *ThGen*_*st*_	Takes the value of one if, in Sweden, there existed a generic version of a substance that was sold in month *t* with the same five-digit ATC code as product *i*.
*N* _ *Th*_*it*_	The number of pharmaceutical substances with the same five-digit ATC code and with locally sourced drugs sold by firms other than the seller of product *i* in month *t*.
*lnN* _ *Th*_*it*_	Natural logarithm of *N* _ *Th*_*it*_ when *N* _ *Th*_*it*_ > 0, but equal to zero when *N* _ *Th*_*it*_ = 0.
*N* _ *ThGen*_*st*_	Number of substances with generic versions and the same five-digit ATC code as product *i* for which generic as product *i* that was sold in month *t*.
*lnN* _ *ThGen*_*st*_	Natural logarithm of *N* _ *ThGen*_*st*_ when *N* _ *ThGen*_*st*_ > 0, but equal to zero when *N* _ *ThGen*_*st*_ = 0.
*Q*_*st*_	The number of defined daily doses sold of products with substance *s* in month *t*.
*lnQ*_*st*_	Natural logarithm of *Q*_*st*_.

The variable *D_PiSubstance*_*st*_ is an indicator that takes the value of 1 if one or more parallel-imported products with the same substance as product *i* were sold in Sweden in month *t*. *D_PiE*_*it*_ is also an indicator, but it only takes the value of 1 if at least one parallel imported product exchangeable with product *i* was sold in Sweden in month *t*. In accordance with the substitution rules, an exchangeable product was defined as a drug with the same active substance, form of administration, strength, and nearly identical package size.

The variable *lnN_PiSubstance*_*st*_ was defined as the natural logarithm of the number of parallel traders selling products with the same substance when *N_PiSubstance*_*st*_ is strictly positive, and takes the value of 0 otherwise. The variable *lnN_PiE*_*it*_ has the corresponding definition for the number of parallel traders selling exchangeable products.^7^ As the natural logarithm of one is zero, *lnN_PiSubstance*_*st*_ and *lnN_PiE*_*it*_ do not change values when the numbers of parallel traders selling products with the same substance and the number of traders selling exchangeable products, respectively, change from zero to one. Therefore, the coefficients for *D* _ *PiSubstance*_*st*_ and *D_PiE*_*it*_ capture the effects of the first parallel trader within the same substance and exchange groups, respectively, whereas the coefficient for *lnN* _ *PiSubstance*_*st*_ and *lnN_PiE*_*it*_ capture the effects of variations in strictly positive numbers of parallel traders.

The variables *D* _ *Th*_*st*_ − *lnN* _ *ThGen*_*st*_ were included to control for competition from firms that sold therapeutic alternatives, that is, products with other pharmaceutical substances that are intended for the same or similar medical diagnoses. *D* _ *Th*_*st*_ takes the value of 1 if at least one other firm sold a locally sourced product with the same five-digit ATC code in month *t*. If there was a generic version of at least one of these substances, also *D* _ *ThGen*_*st*_ takes the value of 1. The variable *N* _ *Th*_*it*_ (not included in the specification) was defined as the number of pharmaceutical substances with the same five-digit ATC code and with locally sourced drugs sold by firms other than the seller of product *i* during month *t*. *lnN* _ *Th*_*it*_ is the natural logarithm of *N* _ *Th*_*it*_ for strictly positive values of this variable and is otherwise 0. Lastly, *lnN* _ *ThGen* was defined as the natural logarithm of the number of therapeutic alternatives for which generic versions exist when this variable is strictly positive, and takes the value of 0 otherwise.

The eight competition variables were all instrumented with their first lags and with *lnQ*_*s*, *t* − 3_, which is the natural logarithm of the quantity of substance *s* sold in month *t* − 3.^8^ Producers have good information about the values of these instruments when, at the end of *t* − 2, they set their prices for month *t*. For the first eight instruments, the reason for this is that the prices of all products that can be sold within the benefit scheme in month *t* − 1 are announced in the first half of month *t* − 2. Hence, producers can observe how many potential competitors they will have in month *t* − 1 and can, based on this, predict the competition they will face in month *t*. Regarding *lnQ*_*s*, *t* − 3_, IMS/IQVIA had delivered sales data for month *t* − 3 to its customers when prices from month *t* were set. The validity of the instruments is discussed and analyzed in the Appendix. Lastly, month and product fixed effects (*η*_*t*_ and *μ*_*i*_) were included in the specification, and the error terms were allowed to be correlated within substances.

To study if the functional form of the preferred specification was too restrictive, we also estimated a specification where *D* _ *PiSubstance*_*st*_, *D* _ *PiE*_*it*_, *lnN* _ *PiSubstance*_*st*_, and *lnN* _ *PiE*_*it*_ were replaced by ten indicator variables for number of parallel trades within the substance and exchange group, respectively. In this specification, the lags of the ten indicator variables were used as instruments instead of *D* _ *PiSubstance*_*s*, *t* − 1_, *D* _ *PiE*_*i*, *t* − 1_, *lnN* _ *PiSubstance*_*s*, *t* − 1_, and *lnN* _ *PiE*_*i*, *t* − 1_; otherwise the specifications were identical to the preferred specification.

Descriptive statistics are presented in Table 2.

**Table 2:** Descriptive statistics

	Oct. 2002–Oct. 2007		Jan. 2011–Dec. 2017			
	Mean	SD	Mean	SD	Min	Max
*P*_*it*_	1462.75	4594.54	3762.37	12,019.43	6.31	290,670.50
*D* _ *PiSubstance*_*st*_	0.24	0.43	0.46	0.50	0	1
*D* _ *PiE*_*it*_	0.11	0.32	0.25	0.43	0	1
*N* _ *PiSubstance*_*st*_	0.62	1.36	1.52	2.11	0	11
*N* _ *PiE*_*it*_	0.24	0.82	0.61	1.34	0	9
*D* _ *Th*_*st*_	0.82	0.39	0.84	0.37	0	1
*D* _ *ThGen*_*st*_	0.53	0.50	0.54	0.50	0	1
*N* _ *Th*_*it*_	2.93	2.50	3.23	3.86	0	28
*N* _ *ThGen*_*st*_	0.89	1.15	1.11	1.41	0	8
*Q*_*st*_ (in millions)	12.91	66.71	9.34	44.40	0.00	85,300.00

*Note*: The number of observations is 132,008 for the first dataset and 101,489 for the second dataset. See Table 1 for variable definitions

## Regression results

The estimation results for the preferred specification are presented in Table 3, while model checks, results from ordinary least square regressions, and robustness analyses are presented in the Appendix. The results for the lag of the dependent variable (*lnP*_*i*, *t* − 1_) show that prices reacted slowly to changes in competition. Taking one minus the coefficient for *lnP*_*i*, *t* − 1_ and multiplying by 100 reveals that only 4 and 8% of the long-term effects were realized immediately in the two sample, respectively.

**Table 3:** Estimation results for *lnP*_*it*_

	*Discounts forbidden*	*Discounts allowed*
	Oct. 2002–Oct. 2007	Jan. 2011–Dec. 2017
*lnP*_*i*, *t* − 1_	0.9568***	0.9171***
	(0.0055)	(0.0174)
*D* _ *PiSubstance*_*st*_	−0.0017***	−0.0012
	(0.0006)	(0.0009)
*D* _ *PiE*_*it*_	−0.0012*	− 0.0003
	(0.0007)	(0.0005)
*lnN* _ *PiSubstance*_*st*_	0.0001	−0.0002
	(0.0004)	(0.0008)
*lnN* _ *PiE*_*it*_	−0.0010*	0.0009
	(0.0006)	(0.0008)
*D* _ *Th*_*st*_	−0.0001	0.0005
	(0.0008)	(0.0019)
*D* _ *ThGen*_*st*_	−0.0007	0.0002
	(0.0006)	(0.0012)
*lnN* _ *Th*_*it*_	−0.0012	−0.0026**
	(0.0012)	(0.0012)
*lnN* _ *ThGen*_*st*_	0.0010	−0.0011
	(0.0007)	(0.0015)
$d{lnP}_i^{\ast }/ dD\_{PiSubstance}_{st}^{\ast }$	−0.0601***	−0.0128
	(0.0151)	(0.0116)
Observations	119,945	90,228
R^2^	0.9183	0.8797
K-P rk LM	72.9704	65.2018
K-P rk LM, *p*-value	0.0000	0.0000
Hansen J, *p*-value	0.1293	0.1792

*Note*: See Table 1 for variable definitions. The specifications include product-specific fixed effects and 58 and 81 indicator variables for months, respectively. In the first-stage regressions, data from Oct. 2002–Oct. 2007 and Jan. 2011–Dec. 2017 were used. K-P rk LM refers to the Kleibergen-Paap rk LM statistic, which indicates the strength of the instruments. The null hypothesis in the K-P test is that the model is under-identified. The null hypothesis for the Hansen J test is that the instruments are valid, i.e., uncorrelated with the error term. Standard errors, robust to correlations within substances, are given in parentheses. ***, **, and * indicate that the coefficient is significantly different from zero on the 1, 5 and 10% significance levels, respectively. The estimation results for the indicator variables for months and for the first-stage regressions are available on request. In short, the first-stage regressions show that their own lag is the strongest instrument for each of the eight competition variables, with t-values ranging from 12 to 143 and point estimates from 0.67 to 0.93, and the R^2^ for the first stage regressions range from 0.49 to 0.89

The coefficients for the eight competition variables show their short-term effects, whereas dividing them by one minus the coefficient for *lnP*_*i*, *t* − 1_ yields their long-term effects. To obtain the exact effect in percentage terms, the formula 100 ∗ [exp(*B*) − 1] should be applied, in which *B* is the coefficient estimate, or long-term estimate, of interest.

For the first study period, the estimates for *D* _ *PiSubstance*_*st*_ and *lnP*_*i*, *t* − 1_ show that the first parallel trader selling products with the same active substance, but which were not exchangeable with product *i*, reduced the price of product *i* by 0.17% in the short-term and 3.9% [≈0.17/(1–0.9568)] in the long term. If the parallel trader instead sold an exchangeable product, so that *D* _ *PiE*_*it*_ also equaled one, the price fell by an additional 2.7% in the long term. Additional parallel importers only reduced the price if they sold exchangeable products, but in this case the effect was small as well; if the sellers of exchangeable products increased from one to three, the price was reduced by 2.2% in the long term.

The differential $d{lnP}_i^{\ast }/ dD\_{PiSubstance}_{st}^{\ast }$ shows the weighted average long-term effect of facing competition from at least one parallel importer selling the same substance.^9^ Applying the formula 100 ∗ [exp(−0.0601) − 1], the effect equaled a price reduction by 5.83% for the first study period. In comparison, the raw (unweighted) average price reduction equaled 5.47%. The results are similar to those reported for tablets and capsules by Granlund [9]. For example, the long-term effect of a first parallel trader that also sold exchangeable products was estimated to be − 7.0% by Granlund [9], whereas here it was − 6.5%.

Column 3 of Table 3 shows that competition from parallel imports had no significant effect on the list prices of locally sourced products in the study period when discounts were allowed. Also, the weighted average effect of facing competition from at least one parallel importer selling the same substance was at the 5% level significantly smaller in the second study period compared with the first study period.

**Fig. 1:**
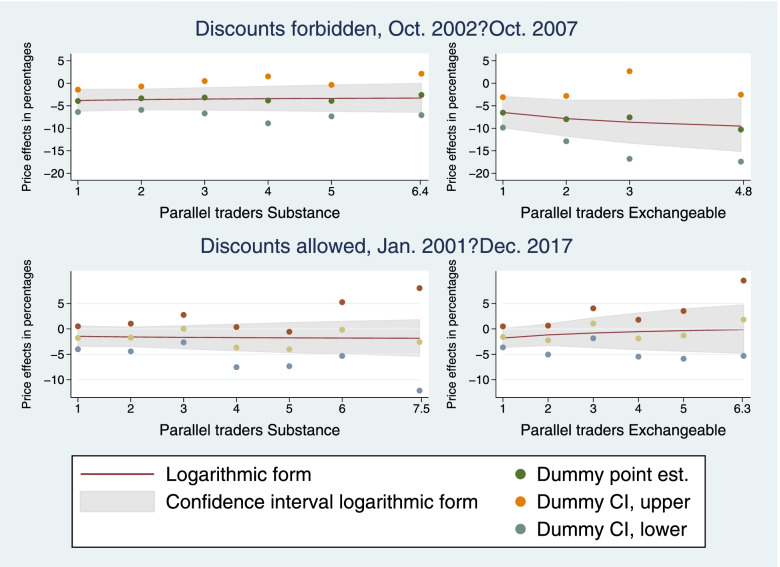
Estimated long-term price effects in percentages of the number of parallel traders selling products with the same active substance and exchangeable products, respectively; comparison of logarithmic-form and flexible-form estimates. The effects in the left panels are plotted holding *N* _ *PiE*_*it*_ at zero, while the effects in the right panels are plotted holding *N* _ *PiSubstance*_*st*_ equal to *N* _ *PiE*_*it*_, see Table 1 for variable definitions. The smooth lines are the long-term effects predicted from the preferred specification of *D* _ *PiSubstance*_*st*_ and *lnN* _ *PiSubstance*_*st*_ (left panels) and of *D* _ *PiSubstance*_*st*_, *D* _ *PiE*_*it*_, *lnN* _ *PiSubstance*_*st*_, and *lnN* _ *PiE*_*it*_ (right panels). The gray area shows the associated 95% confidence intervals. *Dummy point est.* shows the long-term effects of indicator variables for the numbers of *N* _ *PiSubstance*_*st*_ (left panels) and for the numbers of *N* _ *PiSubstance*_*st*_ and *N* _ *PiE*_*it*_ (right panels), and *Dummy CI, upper* and *Dummy CI, lower* show the lower and upper bounds of the associated 95% confidence intervals. These estimates come from an IV specification including indicator variables for the numbers of parallel importers. However, groups with few observations were grouped together to avoid indicators that take the value of one for less than 1% of the observations. The estimates for these merged groups are plotted at the average value of *N* _ *PiSubstance*_*st*_ and *N* _ *PiE*_*it*_ in each merged group, respectively

Figure 1 shows that similar results were obtained when instead using a more flexible specification with indicator variables for the numbers of parallel importers. However, for the period when discounts were forbidden, the confidence intervals were larger when indicator variables were used, which resulted in some estimates not being statistically significantly different from zero. For the period when discounts were allowed, the most notable difference was that the indicator variable for five parallel traders selling products with the same active substance was significantly different from zero, while no significant estimate was obtained using the preferred specification.

Regarding the results for therapeutic competition, there were no statistically significant price effects in the first study period. For the second study period, the estimates for *D* _ *Th*_*st*_ and *D* _ *ThGen*_*st*_ imply that there was no significant price effect from a first therapeutic competitor, but the estimates for *lnN* _ *Th*_*it*_ and *lnN* _ *ThGen*_*st*_ indicate that prices fell with additional competitors.

## Total savings of parallel imports when discounts were forbidden

The pharmaceutical costs in the absence of parallel imports were calculated by multiplying the sold quantity of all products (both locally sourced products and parallel imports) by the price that locally sourced products would have had in the absence of parallel imports.^10^ The savings are the difference between this cost and the actual cost. The savings were calculated using data from January 2003–October 2007 (i.e., for the period used in the second stage of the IV regressions) and divided into one direct and two indirect parts; one for locally sourced products and one for parallel-imported products.

The direct savings consists of the sum over all parallel-imported products of the number of packages sold multiplied by the price difference between these and their locally sourced counterparts. Parallel imports were on average 9% cheaper than locally sourced products, yielding an average annual direct saving of 231 million SEK (24 million Euros) in 2017 prices.^11^ After discounts were allowed, the gaps in list prices between parallel imports and locally sourced drugs were on average only 0.8%.^12^

The indirect savings for locally sourced products were calculated as the total sales value of locally sourced products for which *D* _ *PiSubstance*_*st*_ = 1, multiplied by 0.0619, which shows in decimal form the estimate of how much more expensive the products would have been if they had not faced competition from parallel imports.^13^ These savings were estimated to average 260 million SEK (27 million EUR) per year. The indirect savings for parallel-imported products were calculated correspondingly, except that we used the sales values that would have existed if these products had been sold at the same price as the locally sourced products. This yielded an estimated average indirect savings for the parallel imports of 161 million SEK (17 million EUR) per year.

**Fig. 2:**
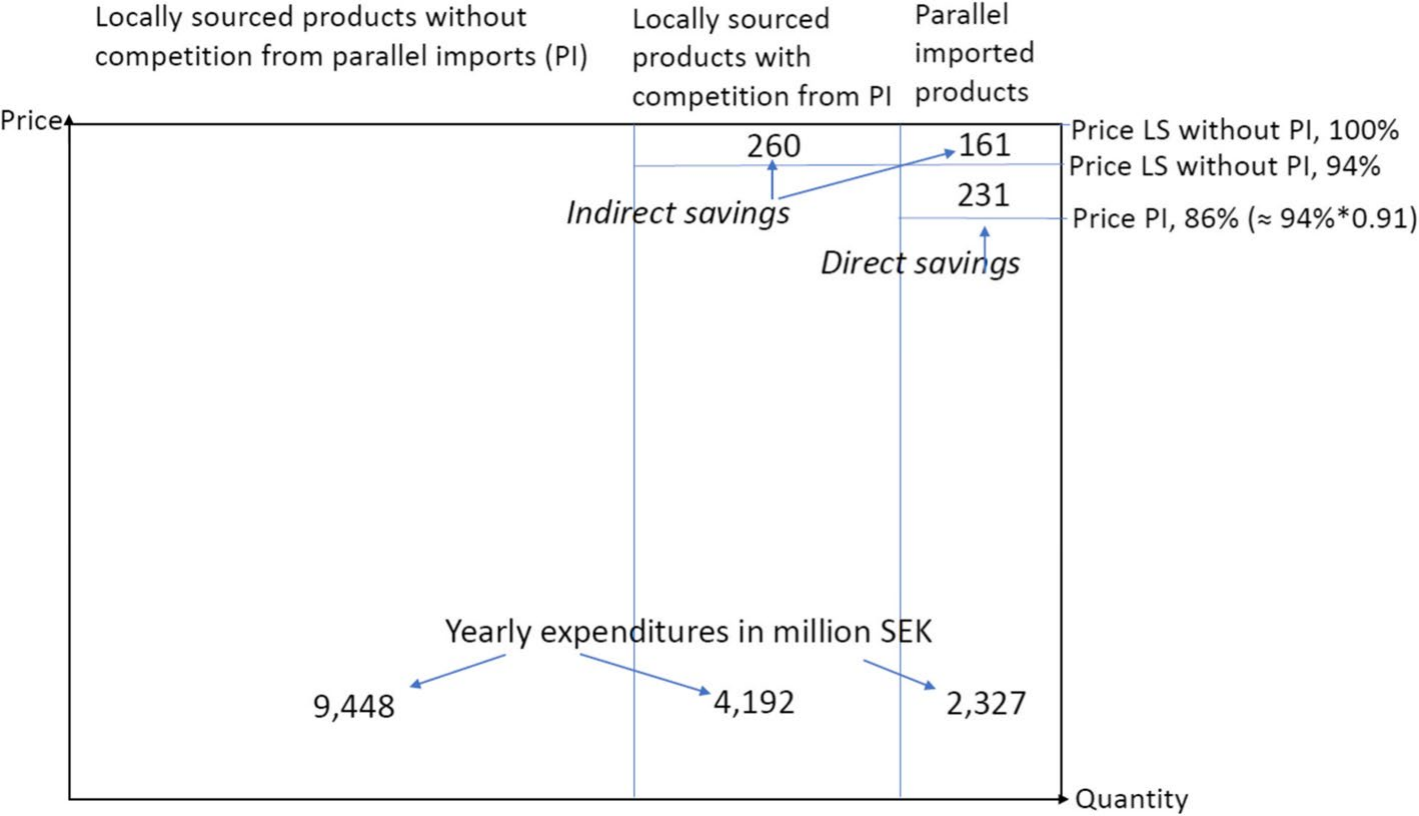
Illustration of average yearly savings for January 2003–October 2007. LS refers to locally sourced products; PI refers to parallel-imported products. The amounts are measured in million SEK of pharmacies’ purchase prices and are expressed in year 2017 prices. In 2017, the average exchange rate was 9.64 SEK for one EUR

The savings are illustrated in Fig. 2 and summarized in Table 4. The rectangle of Fig. 2 illustrates how large the annual expenditure on patent prescription pharmaceutical was estimated to have been without competition from parallel imports. For locally sourced products that did not face competition from parallel imports, no savings occurred. The savings for the other two categories are illustrated in the upper right corner of the figure. All in all, the estimated annual savings generated by parallel imports of pharmaceuticals before discounts were allowed totaled 652 million SEK (≈ 260 + 161 + 231) (68 million EUR). This amounts to 4% of the 16.619 billion SEK that on-patent prescription pharmaceuticals were predicted to have cost without parallel imports.

**Table 4:** Predicted savings with CIs, in millions SEK

	Point estimate	95% CI estimation uncertainty	95% CI PSA
*Direct savings*	231		113–375
*Indirect LS*	260	130–394	134–424
*Indirect PI*	161	81–245	81–271
*Indirect LS + PI*	421	211–638	223–679
*Direct + indirect PI*	393	312–476	238–577
*Total savings*	652	442–869	391–968

*Note*: The asymmetry in the CIs in Column 3 is explained by the concavity of C/(100 - C) (described in footnote 13), which is only partly offset by the convexity of C = 100*[exp(B)-1]. For the PSA CIs, the randomness of the Monte Carlo simulations is also a source of asymmetry. In 2017, the average exchange rate was 9.64 SEK for one EUR

In Table 4, Column 2 reports the point estimates, and Column 3 reports the 95% confidence intervals (95% CI) that reflect uncertainty in the estimated price effects of competition from parallel imports only. The last column reports the CIs from a probabilistic sensitivity analysis (PSA) that also accounted for variability in competition from parallel imports, market shares, and relative prices. The CIs are further described and discussed in the Appendix.

## Discussion and conclusions

When discounts were forbidden, parallel imports had a market share of 16% and were on average 9% cheaper than locally sourced drugs, which directly reduced the cost for on-patent pharmaceuticals by 1.4%. Additionally, we estimated that parallel imports reduced the prices of products with the same substance by, on average, 6% in the long-term. Combining this with the share facing competition from parallel imports indicates that, in total, parallel imports reduced the cost of on-patent pharmaceuticals by 4%.

The estimated price-effects of competition from parallel imports are significantly lower than reported by previous studies [3–5] that used possible endogenous instruments, such as exchange rates. A main advantage of the dynamic model used in this study is that the lagged dependent variable controls for previous price shocks, which makes lagged values of the competition variables valid instruments for the current values. This provides enough strong instruments to estimate the price effects of competition from parallel imports on both the extensive and intensive margin and to do this using flexible specifications. A drawback of the dynamic model is that including a lag of the dependent variable in models with fixed effects can result in bias [16]. As described in the Appendix, this bias is expected to be very small for datasets with high numbers of time periods, such as those used in this study. For short study periods, researchers might have to address this problem by using methods such as an Arellano-Bond estimator [17], which can, in turn, make it difficult to find strong instruments for the competition variables.

For the period when discounts were forbidden, we found no statistically significant effects on list prices despite narrow confidence intervals. This strengthens the conjecture that the preference of locally sourced product sellers to compete with parallel imports by giving discounts to pharmacies is a main reason that previous studies [6–8] have not found significant effects on list prices when discounts were legal. Some advantages of using discounts are that they can be quickly reverted and do not affect the maximum prices producers are allowed to charge in countries that use external reference pricing [18].

No estimates exist regarding the discounts given by sellers of locally sourced products, but the discounts given by parallel traders have been estimated to be about 470 million SEK per year (49 million EUR).^14^ This is within the CI of savings in the pre-reform period caused by parallel imports having lower prices than their locally sourced counterparts would have had if their prices had not been lowered due to competition from parallel imports (point estimate, 393 million SEK). It is conceivable that allowing discounts had small effects on the total savings caused by parallel imports, but allowed those saving to go to pharmacies rather than to consumers and the pharmaceutical benefit scheme. In the case of Sweden, the government can easily redirect savings from pharmacies to consumers and the pharmaceutical benefit scheme by changing the formula that dictates how high the prices pharmacies charge should be in relation to the list prices they pay when not receiving discounts.

From the results of this study, one cannot draw any conclusion on whether it is preferable to allow or forbid sellers of on-patent pharmaceutical to give discounts to pharmacies, as the savings caused by parallel imports can be equally large under both conditions. However, the results clearly show that, from the perspective of payers in destination countries, it is beneficial to continue allowing parallel imports. Likewise, introducing rules that are hard to meet for parallel imports—for example that firms selling pharmaceuticals to pharmacies need to have large quantities in stock, as suggested by a Swedish government inquiry—will be costly. From a global welfare perspective, it is harder to draw policy conclusions regarding parallel trade because the savings of consumers and insurances come at the expense of reduced revenues for sellers of locally sourced drugs. To reduce the amount of parallel trade, these sellers might also increase prices and delay launch of new drugs in low-income countries, which could cause welfare losses [22]. Theoretical studies indicate that the total welfare effects of allowing parallel trade with pharmaceuticals are generally ambiguous and partly depend on differences in national pricing rules [23, 24], patients’ preferences [23, 25] and the vertical integration of trading companies [26].

## Figures and Tables

**Fig. 3:**
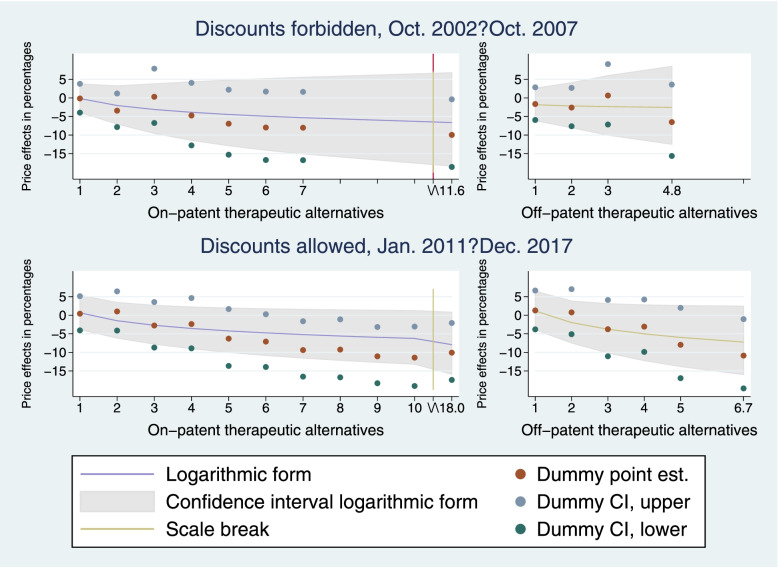
Estimated long-term price effects in percentages of on- and off-patent therapeutic alternatives, respectively; comparison of logarithmic-form and flexible-form estimates. The effects in the left panels are plotted holding *N _ ThGen*_*st*_ at zero, while the effects in the right panels are plotted holding *N _ Th*_*it*_ equal to *N _ ThGen*_*st*_. The smooth lines are the long-term effects predicted from the preferred specification of *D _ Th*_*st*_ and *lnN _ Th*_*it*_ (left panels) and of *D _ Th*_*st*_, *D _ ThGen*_*st*_, *lnN _ Th*_*it*_, and *lnN _ ThGen*_*st*_ (right panels). The gray area shows the associated 95% confidence intervals. Dummy point est. shows the long-term effects of indicator variables for the numbers of *N _ Th*_*it*_ (left panels) and for the numbers of *N _ Th*_*it*_ and *N _ ThGen*_*st*_ (right panels), and Dummy CI, upper and Dummy CI, lower show the upper and lower bounds of the associated 95% confidence intervals. These estimates come from an IV specification including indicator variables for the numbers of therapeutic alternatives. However, groups with few observations were grouped together to avoid indicators that take the value of one for less than one percent of the observations. The estimates for these merged groups are plotted at the average value of *N _ Th*_*it*_ and *N _ ThGen*_*st*_ in each merged group, respectively. In the left panels, the x-axes are halted after *N _ Th*_*it*_* = 10* of space concerns

## Data Availability

The datasets generated for the study are available from the author on reasonable request, except that data on quantities cannot be released without consent from IQVIA.

## Appendix

### Model checks, OLS results, and robustness analyses

Because non-stationarity can result in spurious results, we tested for this using the ADF version of a Fisher-type test, as implemented in xtunitroot in STATA. This test allows for unbalanced panels with gaps within panels and rejected non-stationarity of *lnP*_*it*_ at the 1 % level for both samples. We included panel means and time trends and tested for non-stationarity both with one lag and without lags.

The instruments used in the two-stage least squares estimations were made valid by including the lag of the dependent variable. This lag controls for previous price changes, which makes the error term dependent only on the current price shock. Therefore, the exclusion restriction for the instrument *Q*_*s*, *t* − 3_ should be fulfilled as previous quantities should have no effect on the current price shock, or in other words, *Q*_*s*, *t* − 3_ should not have any additional effect on prices in month *t* after controlling for prices in month *t* − 1. Also, if parallel traders and therapeutic competitors cannot predict the price shock in month *t*, and hence not *ε*_*it*_, their decisions to be active in the Swedish market in month *t* − 1 cannot be a function of *ε*_*it*_. Therefore, if competitors cannot predict price shocks of locally sourced products, the one-month lags of the competition variables should not be a function of *ε*_*it*_ and therefore be exogenous. As the lags of the competition variables should not have any independent effect on the dependent variable except through the endogenous variables and the lag of the dependent variable, the exclusions restrictions for them should therefore be fulfilled.

However, competitors might be able to predict price shocks of locally sourced products if the error-terms are serially correlated. Therefore, we tested for serial correlation up to the third order using the test proposed by Cumby and Huizinga [27]. The null hypothesis of no serial correlation of the second order was rejected at the 5% significance level for the first estimation presented in the paper. Still, the Hansen J test (reported in Table 3) does not reject that the instruments are valid, suggesting that the serial correlation must be small. The first lags of the competition variables would be invalid instrument if firms, when they in month *t* − 3 decides whether or not to have a price for month *t* − 1, can predict the error term for month *t*. Because prices for month *t* − 2 is announced in month *t* − 3, firms can partly do this if they have information about serial correlation of the second, or higher, order, but serial correlation of the first order is not problematic in this respect. Estimations using a generalized linear model accounting for serial correlation up to order three confirmed that the serial correlations of order two and three are small even though three of them are statistically different from zero. In the first and second datasets the estimated correlations are respectively 0.03 and − 0.01 between *ε*_*it*_ and *ε*_*i*, *t* − 2_, and 0.01 and − 0.02 between *ε*_*it*_ and *ε*_*i*, *t* − 3_.

With a correlation of 0.03 between *ε*_*it*_ and *ε*_*i*, *t* − 2_, firms could (if they had estimated models like those presented here) predict 3% of the variation in *ε*_*it*_ when taking decisions that affect the value of the first lags of the competition variables. This could cause a small bias. For example, based on Monte Carlo simulations, Keele and Kelly [28] reported biases of less than 1 % for both the short- and long-term effects when the correlation coefficient is 0.10. The bias would be smaller if the second lags of the competition variables were used as instruments instead of the first lags, because *ε*_*it*_ is less correlated with *ε*_*i*, *t* − 3_ than with *ε*_*i*, *t* − 2_. Tables 5 and 6 therefore report estimation results obtained when using second lags of the competition variables as instruments (specification IV 2). Using second lags reduces the number of observations. Therefore, to separate direct effects of the choice of instruments from the effects of changed sample, we report the results obtained by using first lags on the samples used for specification IV 2 (specification IV 1 s2). To facilitate comparisons, the other specifications presented in Tables 5 and 6 are also estimated on the same samples. The results of specifications IV 2 and IV 1 s2 are similar, which indicates that the choice of instruments had minor effects on the results and confirms that the possible bias is small. Additionally, comparison with the results of Table 3 shows that using slightly smaller samples had no important effect on the results.

Specification IV 1 s2 cl5 only differs from specification IV 1 s2 by allowing error terms to be correlated within therapeutic groups (i.e., 5-digit ATC groups) instead of within substances (i.e., 7-digit ATC groups). Tables 5 and 6 show that the results are robust to this choice of clustering unit. In the first study period, most estimated standard errors are identical up to the fourth decimal. In the second study period, the standard errors change slightly more but do not alter the conclusion that parallel imports had no significant effect on list prices in that period.

Tables 5 and 6 also reports ordinary least squares results for the preferred specifications as well as for a static specification. IV results are not reported for the static specifications because we lack instruments that are valid when the lagged dependent variable is not included. This is because, without the lagged dependent variable, the error term is a function of past price shocks which makes lagged variables of number of competitors invalid as instruments.

As for the results obtained for the dynamic specifications for the first study period, the negative point estimates for the effects of competition from parallel imports become slightly less negative when OLS is used. As a result, the estimate for the differential $d{lnP}_i^{\ast }/ dD\_{PiSubstance}_{st}^{\ast }$, which shows the weighted average long-term effect of competition from at least one parallel importer selling the same substance,^15^ falls by about 1 %age point in absolute size when OLS is used. For the second study period, the estimates for *D* _ *PiSubstance*_*st*_ and *D* _ *PiE*_*it*_ change oppositely when OLS is used instead of IV, but the sum of the estimates for these two variables are nearly unaffected.

The differences between the dynamic and static OLS specifications are larger. The static specification gives significantly positive estimates for *lnN* _ *PiE*_*it*_ in both study periods, indicating that reverse causality (that parallel traders are attracted to exchange groups with high prices) dominates for this variable. This is one indication of that the static OLS specification is not a valid specification in this setting. In the static specification, the estimates for the other variables measuring competition from parallel imports either becomes more positive or is amplified by less than what is required to give the same long-term effects as in the dynamic specification. Specifically, the negative estimates are amplified by less than 1/(1 − *θ*), where *θ* is the parameter for *lnP*_*i*,*t* − 1_ in the dynamic specification. As a result, the differential $d{lnP}_i^{\ast }/ dD\_{PiSubstance}_{st}^{\ast }$ becomes positive for both study periods when the static OLS specification is used. Overall, the results in Table 5 indicate that including the lagged dependent variable (*lnP*_*i*, *t* − 1_) reduces the endogeneity problem, but that it can still be important to instrument potentially endogenous variables.

Simultaneously including fixed effects and a lag of the dependent variable can cause bias. Fortunately, this bias in the estimator for the coefficient of the lagged dependent variable (*θ*) decreases in the number of time periods. According to Nickell [16], the limit of the bias for the parameter *θ* as N approaches infinity can be approximated by –(1 + *θ*)/(T − 1), in which N and T are the number of fixed effects and time periods, respectively. Additionally, for *θ* = 0.9 and when 90% (95%) of the total variance is due to fixed effects, Nerlove [29] found a bias that was just 40% (26%) of the bias suggested by the approximation written above. For the two samples, the fixed effects explain 93 and 96% of the total variation, and the averages of time periods a product is included in the analyses are 42 and 43, respectively. With *θ*-values of 0.96 and 0.92, respectively, this bias would be about − 0.016 and − 0.012, assuming that the bias is 33% (= (40% + 26%)/2) and 26% of –(1 + *θ*)/(T − 1), respectively. Because of the small magnitudes of the expected biases and since we were not able to instrument the explanatory variables when using first-difference transformation, we presented results from estimations in which we have not accounted for this small bias. Using the first-difference transformation and estimators such as the Arellano-Bond estimator gave far less robust and precise estimates than the chosen partial adjustment estimator.

To study if the functional form of the preferred specification is too restrictive to accurately capture the price effects of the numbers of on- and off patent therapeutic alternatives, we have also estimated specifications where *D* _ *Th*_*st*_, *D* _ *ThGen*_*st*_, *lnN* _ *Th*_*it*_, and *lnN* _ *ThGen*_*st*_ were replaced by 12 indicator variables of *N* _ *Th*_*it*_ and *N* _ *ThGen*_*st*_ in the first study period and 17 indicator variables in the second study period. In these specifications, the lags of the indicator variables were used as instruments instead of *D* _ *Th*_*s*, *t* − 1_, *D* _ *ThGen*_*s*, *t* − 1_, *lnN* _ *Th*_*i*, *t* − 1_, and *lnN* _ *ThGen*_*s*, *t* − 1_. Figure 3 show the predicted price-effects of number of therapeutic alternatives from these specifications together with the predictions from the preferred semi-logarithmic specification. The predictions are similar, but while the predictions from the preferred specification are not significantly different from zero in the ranges of the graphs, the flexible specifications indicate that the price-effects of number of therapeutic alternatives are statistically significant when the number of alternatives is very high; more precisely, when the number of on-patent alternatives are eight or more in the first study period and seven or more in the second study period, and when the number of off-patent therapeutic alternatives exceeds five in the second study period. Lastly, results not presented in figures or tables show that the estimates for the price effects of competition from parallel imports were nearly identical for the flexible specifications and the preferred specification.

### Discussion about the confidence intervals reported in Table 4

Table 4 in the paper lists the point estimates of the savings together with 95% confidence intervals. The confidence intervals that only reflect the uncertainty in the estimated price effects of facing competition from parallel imports are relevant if one knows: i) the sales values for locally sourced products facing competition from parallel imports, ii) the extent of the competition these products face, and iii) market shares and relative prices of parallel imports. However, beforehand also these variables are unknown because they depend on, among else, the decision of parallel traders and prescribers, pharmacies policies and patient preferences. Therefore, Column 4 presents confidence intervals from a probabilistic sensitivity analysis (PSA) that also accounts for these sources of uncertainty.

The PSA was performed by making 10,000 independent draws from the distribution of the estimates for the lag of the dependent variable and for the variables describing the extent of competition from parallel imports, and of one of the 61 months in the data. For each month drawn and the following eleven months, direct savings, averages values (weighted with sales) of the four variables describing competition from parallel imports, and markets share of parallel imports were calculated. Together with the draws from the distribution of the estimates, these were used to calculate estimates of yearly savings. To equalize the expected number of times data from each month were used, we treated time in a circular manner meaning that, e.g., if the 61st month was drawn, also data from the first 11 month were used to calculate 12-months values.

Column 3 of Table 4 shows the additive property of the confidence intervals that only accounts for the estimation uncertainty. For example, the sum of the confidence interval for *Indirect LS* and *Indirect PI* equals the confidence interval for *Indirect LS + PI*, except from rounding effects, and the widths of the confidence intervals for *Total Savings* and *Indirect LS + PI* are equal because *Direct savings* is not affected by the estimation uncertainty. This property does not hold for the confidence intervals from the probabilistic sensitivity analysis. The reason is that uncertainties in some variables canceled out at least partially. For example, variation in the market shares of parallel imports affects the PSA confidence intervals for both *Indirect LS* and *Indirect PI*, but do not affect the PSA confidence interval for *Indirect LS + PI*.

**Table 5:** Robustness checks for Oct. 2002–Oct. 2007, estimation results for *lnP*_*it*_

	IV 2	IV 1 s2	IV 1 s2 cl5	OLS s2	Static OLS s2
*lnP*_*i*, *t* − 1_	0.9568***	0.9568***	0.9568***	0.9568***	
	(0.0055)	(0.0055)	(0.0057)	(0.0055)	
*D* _ *PiSubstance*_*st*_	−0.0019***	− 0.0017***	− 0.0017***	− 0.0015***	−0.0106**
	(0.0007)	(0.0006)	(0.0006)	(0.0005)	(0.0045)
*D* _ *PiE*_*it*_	−0.0012	−0.0012*	− 0.0012	−0.0010*	0.0068
	(0.0008)	(0.0007)	(0.0007)	(0.0005)	(0.0049)
*lnN* _ *PiSubstance*_*st*_	0.0000	0.0001	0.0001	0.0001	0.0057
	(0.0004)	(0.0004)	(0.0004)	(0.0003)	(0.0057)
*lnN* _ *PiE*_*it*_	−0.0012*	−0.0011*	−0.0011*	− 0.0007	0.0298***
	(0.0007)	(0.0006)	(0.0006)	(0.0005)	(0.0110)
*D* _ *Th*_*st*_	−0.0002	−0.0001	− 0.0001	− 0.0002	0.0040
	(0.0010)	(0.0009)	(0.0009)	(0.0007)	(0.0099)
*D* _ *ThGen*_*st*_	−0.0012**	−0.0007	− 0.0007	− 0.0002	− 0.0127***
	(0.0006)	(0.0006)	(0.0006)	(0.0003)	(0.0045)
*lnN* _ *Th*_*it*_	−0.0003	−0.0012	− 0.0012	−0.0004	− 0.0049
	(0.0013)	(0.0012)	(0.0013)	(0.0010)	(0.0127)
*lnN* _ *ThGen*_*st*_	0.0010	0.0009	0.0009	0.0007	−0.0199**
	(0.0008)	(0.0007)	(0.0007)	(0.0006)	(0.0095)
$d{lnP}_i^{\ast }/ dD\_{PiSubstance}_{st}^{\ast }$	−0.0671***	−0.0603***	− 0.0603***	− 0.0496***	0.0067
	(0.0165)	(0.0151)	(0.0156)	(0.0129)	(0.0087)
Observations	119,058	119,058	119,058	119,058	119,058
R^2^	0.9181	0.9181	0.9181	0.9181	0.0201
K-P rk LM	69.1940	72.9573	41.6238		
K-P rk LM, *p*-val.	0.0000	0.0000	0.0000		
Hansen J, *p*-value	0.1496	0.1346	0.1439		

*Note*: See Table 1 for variable definitions. The specifications include product-specific fixed effects and indicator variables for year × month combinations. In the first-stage regressions, data from Oct. 2002–Oct. 2007 and Jan. 2011–Dec. 2017 were used. K-P rk LM refers to the Kleibergen-Paap rk LM statistic, which indicates the strength of the instruments. The null hypothesis in the K-P test is that the model is under-identified. The null hypothesis for the Hansen J test is that the instruments are valid, i.e., uncorrelated with the error term. Standard errors, robust to correlations within substances, are given in parentheses. ***, **, and * indicate that the coefficient is statistically significant different from zero on the 1, 5 and 10% significance levels, respectively. The estimation results for the indicator variables for year × month combinations and for the first-stage regression are available on request

**Table 6:** Robustness checks for Jan. 2011–Dec. 2017, estimation results for *lnP*_*it*_

	IV 2	IV 1 s2	IV 1 s2 cl5	OLS s2	Static OLS s2
*lnP*_*i*, *t* − 1_	0.9193***	0.9194***	0.9194***	0.9194***	
	(0.0177)	(0.0176)	(0.0202)	(0.0176)	
*D* _ *PiSubstance*_*st*_	−0.0011	− 0.0012	− 0.0012	−0.0007	− 0.0069
	(0.0010)	(0.0009)	(0.0009)	(0.0005)	(0.0070)
*D* _ *PiE*_*it*_	−0.0005	−0.0003	− 0.0003	−0.0006	0.0022
	(0.0006)	(0.0005)	(0.0005)	(0.0004)	(0.0046)
*lnN* _ *PiSubstance*_*st*_	−0.0003	−0.0001	− 0.0001	−0.0001	0.0018
	(0.0009)	(0.0008)	(0.0009)	(0.0006)	(0.0087)
*lnN* _ *PiE*_*it*_	0.0010	0.0009	0.0009	0.0009	0.0161***
	(0.0009)	(0.0008)	(0.0007)	(0.0007)	(0.0061)
*D* _ *Th*_*st*_	0.0021	0.0010	0.0010	−0.0000	−0.0235
	(0.0020)	(0.0017)	(0.0018)	(0.0011)	(0.0209)
*D* _ *ThGen*_*st*_	0.0004	0.0003	0.0003	0.0000	0.0115
	(0.0012)	(0.0012)	(0.0014)	(0.0010)	(0.0119)
*lnN* _ *Th*_*it*_	−0.0037**	−0.0025**	− 0.0025*	−0.0011	− 0.0183*
	(0.0015)	(0.0012)	(0.0014)	(0.0009)	(0.0101)
*lnN* _ *ThGen*_*st*_	−0.0010	−0.0012	− 0.0012	−0.0011	− 0.0091
	(0.0017)	(0.0015)	(0.0017)	(0.0013)	(0.0128)
$d{lnP}_i^{\ast }/ dD\_{PiSubstance}_{st}^{\ast }$	−0.0152	−0.0132	− 0.0132	−0.0085	0.0055
	(0.0122)	(0.0112)	(0.0128)	(0.0107)	(0.0104)
Observations	89,292	89,292	89,292	89,292	89,292
R^2^	0.8830	0.8831	0.8831	0.8831	0.0122
K-P rk LM	72.1080	65.6942	24.1794		
K-P rk LM, *p*-val.	0.0000	0.0000	0.0000		
Hansen J, *p*-value	0.1830	0.1639	0.1950		

*Note*: See Table 1 for variable definitions. The specifications include product-specific fixed effects and indicator variables for year × month combinations. In the first-stage regressions, data from October 2002–October 2007 and Jan. 2011–Dec. 2017 were used. K-P rk LM refers to the Kleibergen-Paap rk LM statistic, which indicates the strength of the instruments. The null hypothesis in the K-P test is that the model is under-identified. The null hypothesis for the Hansen J test is that the instruments are valid, i.e., uncorrelated with the error term. Standard errors, robust to correlations within substances, are given in parentheses. ***, **, and * indicate that the coefficient is statistically significant different from zero on the 1, 5 and 10% significance levels, respectively. The estimation results for the indicator variables for year × month combinations and for the first-stage regression are available on request

## Abbreviations

CI: Confidence Intervals
K-P rk LM: Kleibergen-Paap rk LM statistic
PBA: Pharmaceutical Benefits Agency
PSA: Probabilistic sensitivity analysis
SEK: Swedish krona

## Footnotes

1 Several countries use international reference pricing in which Swedish prices influence the highest price that the producers are allowed to charge. For example, the reference prices in Austria, Ireland, and Switzerland are the average of prices in Sweden and 26, 13, and 8 other countries, respectively [1].

2 Vandoros and Kanavos [6] did not discuss the magnitude of their estimates, but their point estimates for the indicator variable *pt* suggest that the presence of parallel imports reduced the prices of locally sourced product by 81 and 38%, respectively. These figures are calculated using the formula 100*[exp(β)-1].

3 Packet sizes are allowed to vary slightly for non-narcotic groups; for example, substitution can be made from a 30-pill package to a package in the 28–32-pill range. For the 3% of observations classified as narcotic drugs, exactly the same packet size is required. Parallel imports usually have exactly the same package size as the locally sourced product. For locally sourced products that are exchangeable to at least one parallel imports, all parallel imported substitutes had exactly the same package size in 85% of cases in the first dataset and 79% in the second dataset. It is rare that locally sourced on-patent products are exchangeable with each other and share substitutes, but this is the case for 5% of the locally sourced observations in the first dataset and 3% in the second dataset.

4 For off-patent pharmaceuticals, the cheapest substitute declared by the seller to be available in Sweden throughout the month is declared to be the product of the month, and this product is considered available at all pharmacies. In other words, consumers opposing substitution for off-patent pharmaceuticals must pay the price difference between the prescribed product and the product of the month, irrespective of whether it is in stock at the pharmacy. As a result, pharmacies in general have the products of the month in stock.

5 Note that the latter data are for all products available in Sweden and that the products’ characteristics are identical throughout the country, so no regional data was used in this study.

6 In January 2008, the public inquiry [13] published their suggestions to allow discounts to pharmacies. A reference group with stake holders were connected to the inquiry and likely knew the final suggestions by the inquiry a few months before it was published.

7 In the first dataset, 382 observations were not used in the regressions, as *lnN_PiE*_*it*_ could not be defined because of missing information on package size for at least one product with the same substance, strength, and form of administration.

8 The first dataset included information on the defined daily dozes for 83% of observations, which was then used to create the quantity variable. For the remaining observations, we used the sum over products of number of pills sold of each product multiplied by the strength of each pill. This yielded values that are proportional to daily doses, which is all that is needed for using variations in *lnQ*_*st*_ over time as a proxy for market growth.

9 The differential $d{lnP}_i^{\ast }/ dD\_{PiSubstance}_{st}^{\ast }$ was defined as (*β*_1_ + *m*_2_*β*_2_ + *m*_3_*β*_3_ + *m*_4_*β*_4_)/(1 − *θ*), in which *m*_2_, *m*_3_, and *m*_4_ are the within-sample weighted means of *D* _ *PiE*_*it*_, *lnN* _ *PiSubstance*_*st*_, and *lnN* _ *PiE*_*it*_, respectively, when *D* _ *PiSubstance*_*st*_ = 1.

10 This means that the total quantity (including both parallel imports and locally sourced drugs) are assumed to be unaffected by the price. If this assumption does not hold, the saving should only be interpreted as an estimate of how much more the sold pharmaceutical quantities would have cost without parallel imports. If, instead, a price elasticity of 0.2% is assumed (as in [15]), parallel imports would have been estimated to have reduced the expenditures by 80% of the figures reported in this paper. However, in this case, parallel imports would have also resulted in consumer surplus from additional quantities used. As reported by Kanavos and Costa-Font [7], estimates on the demand elasticity for prescription pharmaceuticals range from close to 0 to − 0.33.

11 In 2017, the average exchange rate was 9.64 SEK for one EURO. For 17% of the observations of parallel-imported products, locally sourced products with the same active substance, form of administration, strength, and package size were not available in the same month. For these observations, the relative price has been assumed to equal the weighted average relative price of parallel-imported products in this month. As weight, we used the product of the number of sold packages of the parallel imported product and the price of the locally sourced product, i.e., the sales values the parallel imports would have had if they had been sold at the same price as the locally sourced products.

12 Allowing for correlation within substances, the standard error for the price difference is only 0.3%, implying that 0.8% is statistically significantly different from zero.

13 The value 0.0619 is calculated with the equation C/(1 - C), in which C = 0.0583 is the weighted average long-term reduction in prices resulting from competition from at least one parallel imported product, as reported in the previous section. The formula reflects that a price cut of 5.83% must be followed by a price increase of 6.19% to be fully off-set.

14 Our own calculations based on [19–21].
