# Correction: Direct and indirect savings from parallel imports in Sweden

**DOI:** 10.1186/s13561-022-00408-5

**Published:** 2022-12-14

**Authors:** David Granlund

**Affiliations:** Department of Economics, Umeå University, Umeå, Sweden

**Correction: Health Econ Rev 12, 46 (2022)**

10.1186/s13561-022-00391-x

Following publication of the original article [1], the author reported errors in Figs. 1 and 3. The year “2001” in Fig. 1 was incorrectly written instead of 2011; and dashes “–” were converted to question marks “?” in two places in all headings of Fig. 1 and 3.

The correct figures are given below.

**Fig. 1:**
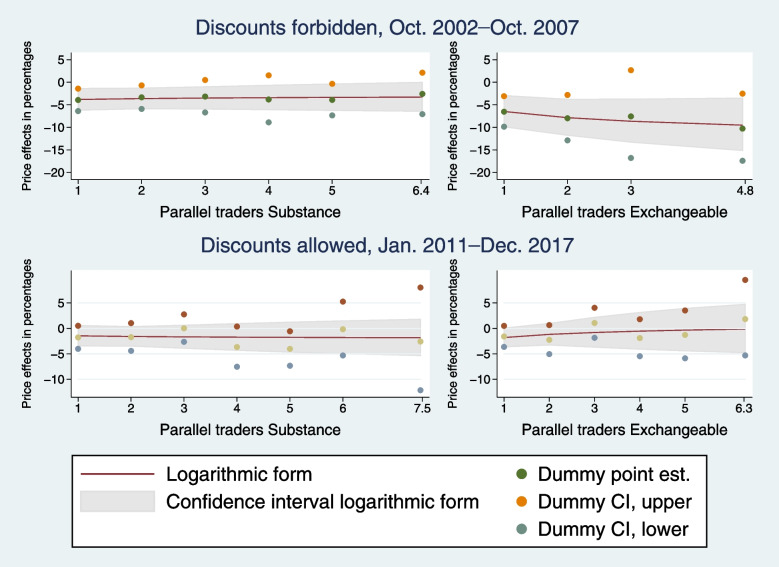
Estimated long-term price effects in percentages of the number of parallel traders selling products with the same active substance and exchangeable products, respectively; comparison of logarithmic-form and flexible-form estimates. The effects in the left panels are plotted holding *N* _ *PiE*_*it*_ at zero, while the effects in the right panels are plotted holding *N* _ *PiSubstance*_*st*_ equal to *N* _ *PiE*_*it*_, see Table 1 for variable definitions. The smooth lines are the long-term effects predicted from the preferred specification of *D* _ *PiSubstance*_*st*_ and *lnN* _ *PiSubstance*_*st*_ (left panels) and of *D* _ *PiSubstance*_*st*_, *D* _ *PiE*_*it*_, *lnN* _ *PiSubstance*_*st*_, and *lnN* _ *PiE*_*it*_ (right panels). The gray area shows the associated 95% confidence intervals. *Dummy point est.* shows the long-term effects of indicator variables for the numbers of *N* _ *PiSubstance*_*st*_ (left panels) and for the numbers of *N* _ *PiSubstance*_*st*_ and *N* _ *PiE*_*it*_ (right panels), and *Dummy CI, upper* and *Dummy CI, lower* show the lower and upper bounds of the associated 95% confidence intervals. These estimates come from an IV specification including indicator variables for the numbers of parallel importers. However, groups with few observations were grouped together to avoid indicators that take the value of one for less than 1% of the observations. The estimates for these merged groups are plotted at the average value of *N* _ *PiSubstance*_*st*_ and *N* _ *PiE*_*it*_ in each merged group, respectively

**Fig. 3:**
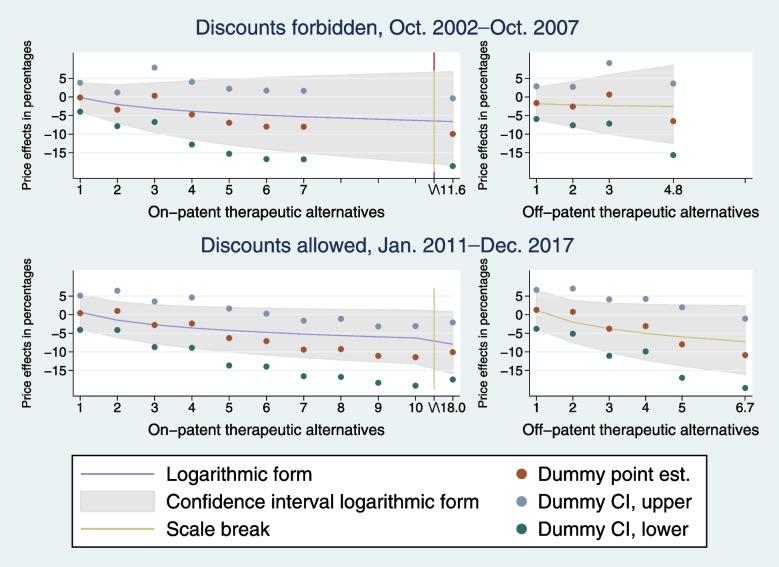
Estimated long-term price effects in percentages of on- and off-patent therapeutic alternatives, respectively; comparison of logarithmic-form and flexible-form estimates. The effects in the left panels are plotted holding *N _ ThGen*_*st*_ at zero, while the effects in the right panels are plotted holding *N _ Th*_*it*_ equal to *N _ ThGen*_*st*_. The smooth lines are the long-term effects predicted from the preferred specification of *D _ Th*_*st*_ and *lnN _ Th*_*it*_ (left panels) and of *D _ Th*_*st*_, *D _ ThGen*_*st*_, *lnN _ Th*_*it*_, and *lnN _ ThGen*_*st*_ (right panels). The gray area shows the associated 95% confidence intervals. Dummy point est. shows the long-term effects of indicator variables for the numbers of *N _ Th*_*it*_ (left panels) and for the numbers of *N _ Th*_*it*_ and *N _ ThGen*_*st*_ (right panels), and Dummy CI, upper and Dummy CI, lower show the upper and lower bounds of the associated 95% confidence intervals. These estimates come from an IV specification including indicator variables for the numbers of therapeutic alternatives. However, groups with few observations were grouped together to avoid indicators that take the value of one for less than one percent of the observations. The estimates for these merged groups are plotted at the average value of *N _ Th*_*it*_ and *N _ ThGen*_*st*_ in each merged group, respectively. In the left panels, the x-axes are halted after *N _ Th*_*it*_ *= 10* of space concerns
